# Examining the relationships between trust in providers and information, mistrust, and COVID-19 vaccine concerns, necessity, and intentions

**DOI:** 10.1186/s12889-022-14399-9

**Published:** 2022-11-07

**Authors:** Lillie D. Williamson, Adati Tarfa

**Affiliations:** 1 Department of Communication Arts, University of Wisconsin-Madison, 6050 Vilas Hall, 821 University Ave, Madison, WI 53706 USA; 2 School of Pharmacy, University of Wisconsin-Madison, Madison, WI USA

**Keywords:** COVID-19, Trust, Medical mistrust, Vaccine intentions

## Abstract

To facilitate maximum uptake of the COVID-19 vaccine, the roles of medical trust and mistrust of healthcare professionals must be examined. Previous work suggests that trust and mistrust may have differential impacts on vaccination intention via vaccine necessity and concerns. Multigroup structural equation modeling was utilized to test whether vaccine necessity and concerns mediated the associations between trust in providers and health information, mistrust of providers, and willingness to get the COVID-19 vaccine. The model was found to be invariant across Black and White respondents. Trust in providers and trust in healthcare information exerted indirect effects on intentions through vaccine necessity, while mistrust of providers exerted indirect effects through vaccine concerns. Unlike previous work, the forms of trust did not influence vaccine concerns. The findings have implications for future communication efforts from healthcare professionals and health messengers.

As of April 2022, over six million individuals worldwide have succumbed to COVID-19 [1], including over 977,000 individuals in the United States [2]. The gravity of this situation has rightfully raised questions about ways to quell the massive number of hospitalizations and devastating loss of life brought on by the pandemic. In addition to masking and social distancing, vaccination is one of the other tools public health officials have at their disposal to combat the COVID-19 pandemic as it can minimize the morbidity and mortality associated with the natural spread of COVID-19 [3, 4]. As states, counties, organizations, and businesses lift mask mandates and other COVID-19 mitigation measures [5], the importance of vaccination for public health may become increasingly consequential.

Existing barriers (e.g., inability to take off work to deal with vaccine side effects) may mean that individuals do not ultimately engage in a specific behavior. However, as we work to reduce those barriers, we must also understand individuals’ intentions to engage in the behavior. Behavior change theories (e.g., the theory of planned behavior) indicate that intentions are the best predictor of behavior: individuals who are willing and intend to get vaccinated will be more likely to engage in the behavior itself [6, 7]. Thus, by having a thorough understanding of individuals’ vaccination intentions, insight is gained about how to improve intentions, and in turn, strides can be taken to help improve COVID-19 vaccination rates.

Physicians’ recommendations are one of the strongest correlates of vaccine intentions and uptake amongst patients [8–11]. This has held true in the context of COVID-19; individuals have reported being more likely to receive the vaccine if they thought their healthcare provider would recommend the vaccine [12]. Individuals’ adherence to provider recommendations is predicated on perceptions of the providers; if providers are not seen as trustworthy or there is distrust, there is no reason for recommendations to be followed. As a result, trust in and mistrust of physicians should be consequential perceptions related to vaccination decisions. Trust is often defined as a willingness to be vulnerable and know that one’s needs will be met [13]. In other words, trust in providers encompasses beliefs that one’s needs will be met because the provider is competent and will do what is best for the patient. Trust in providers has been repeatedly established as a factor in a variety of vaccination behaviors [14, 15].

Previous studies of trust and vaccination have focused on trust in providers but perceptions of the information itself may influence vaccination intentions. Trust in a source, generally, and trust in the information provided by a particular source can differ [16]. It is possible, for instance, that individuals distinguish between trust in a messenger (i.e., a healthcare professional) and the message or information itself [17]. Some scholars have suggested that trust in the messenger and trust in the message are related but distinct concepts, that can have reciprocal relationships with one another [18]. An individual may trust in the health information provided but believe the provider is untrustworthy more globally, in other aspects. Previous work on trust related to vaccination has nested trust in information within trust in that particular entity or focused on governmental sources [19, 20]. The current study sought to disaggregate this and examine not only trust in healthcare providers but trust in healthcare information from providers.

As articulated by Jaiswal and Halkitis, medical mistrust is more than the absence of trust, it reflects beliefs and concerns that medical institutions and personnel may actively work against patients’ best interests [21]. In other words, mistrust would speak to beliefs about the negative motives of physicians. Like medical trust, previous scholarship has also established mistrust as a factor in willingness to get vaccinated [22, 23]. However, studies rarely examine the two constructs simultaneously. Some work in contexts outside of vaccination suggests that trust and mistrust have differential impacts on outcomes. For example, previous work found that mistrust of healthcare providers but not trust in healthcare providers predicted medication adherence [24]. To date, to our knowledge, studies have not examined both trust and mistrust in relation to vaccination intentions.

In thinking about how to communicate about vaccination, it is important to not only understand whether trust and mistrust may affect vaccination intentions but why it is these effects occur. Thus, we examine two possible mediators: vaccine necessity and vaccine concerns. From a health communication perspective, both vaccine necessity and vaccine concerns are pertinent antecedents to vaccine intentions, particularly as they represent benefits of (i.e., necessity) and barriers (i.e., concerns) to the behaviors. Previous work has demonstrated the effects that benefits and barriers play in health decision-making [25]. Thus, examining vaccine necessity and concerns, as benefits and barriers of COVID-19 vaccination, respectively, might provide insight into how individuals are deciding whether to get the vaccine.

Pellowski and colleagues found that trust and mistrust had differential impacts on medication concerns and necessity in the context of antiretrovirals [24]. In their study, trust in physicians was positively related to medication necessity and negatively related to medication concerns. Mistrust in physicians, on the other hand, was not related to medication necessity and positively related to medication concerns. While Sars-COV-2 and the human immunodeficiency virus (HIV) are different viruses, the framework for considering the relationship between trust, mistrust, and vaccine concerns and necessity is a useful approach; scholars have pointed to other literature around HIV for insights into approaches to COVID-19 [26, 27].

Previous work has consistently found racial differences in medical trust and mistrust, as well as vaccination behaviors [28–32]. As a result, there may be racial differences in the aforementioned proposed relationships. For Black Americans, there is historical context contributing to their lowered trust and heightened mistrust. Although the United States Public Health Services Syphilis Study at Tuskegee is one of the most salient examples, it is not the only example. This is one event in a long line – from the testing of procedures on unanesthetized enslaved Black women to radiation experiments on Black soldiers [33]. In addition to historical events, the continued personal and vicarious experiences of racism and racial discrimination faced in health care also contribute to medical mistrust [34]. The distribution of these events suggests that these links from medical trust and mistrust to vaccine intentions may be different for Black and White Americans.

Trust and mistrust are relevant constructs in the context of other vaccines, but each vaccine context is different [35, 36]; this study presents an opportunity to determine the extent to which these relationships may also be operating in this context as well. Thus, the current study sought to a) examine the relationships between trust, mistrust, vaccine necessity, vaccine concerns, and vaccine intentions in the context of COVID-19 and b) determine whether these relationships differ across racial groups. Additionally, the study tested vaccine necessity and concerns as part of the mechanism through which trust and mistrust impact vaccination intentions.

## Methods

### Participants and recruitment

Following the study’s approval as exempt by the Institutional Review Board at the University of Wisconsin-Madison (ID # 2020–1541), we conducted a cross-sectional study using the electronic distribution of a questionnaire to a Qualtrics panel in January 2021. Recruitment sought individuals who were at least 18 years of age, self-identified as Black or White, and resided in the United States.

### Procedure

Participants were informed they were being asked to take part in a 15–20-minute survey. The variables reported here are part of a larger study examining the relationships and differences in the measurement of trust, mistrust, and their antecedents (e.g., medical skepticism). The survey also included items related to the current pandemic; in addition to those items presented here, participants were asked about their news consumption and exposure. The present study focuses on aspects of trust, mistrust, vaccine concerns, vaccine necessity, and vaccination intentions. These items were presented before questions about news exposure; thus, these other items should have no effect. Additionally, within relevant study items, the presentation of items was counterbalanced to prevent order effects. For instance, the order in which trust in providers, trust in information, and mistrust of providers were presented was randomized.

### Measures

Study variables were measured via a self-report questionnaire. Unless otherwise noted, participants responded on a five-point Likert scale from 1 (strongly disagree) to 5 (strongly agree).

#### Trust in providers

Trust in providers was assessed using the trust in physician scale [13]. This 10-item measure assesses the perceived trust patients have in the fidelity, confidence, and honesty of physicians, as well as a global assessment of trust. Prior work has found the measure to be highly reliable (α = .93) [13]. During analysis, three items, all of which had been reverse coded, did not load onto the construct. Given previous work regarding reverse-coded items these items were removed [37]. The resulting seven items formed a reliable measure (α = .93).

#### Trust in healthcare information

Based on work by Lee and Hornik, respondents were asked on a 4-point scale (0 = “not at all” to 3 = “a lot”) how much they trust the information from their doctor or other health care professional [38].

#### Mistrust in providers

Mistrust in providers was assessed using the Medical Mistrust Index (MMI) [39]. The seven-item scale asks participants to rate their degree of agreement with items related to mistrust of healthcare organizations. For the current study, we adapted the questions to ask about healthcare providers. For example, in the original scale participants were asked “You ‘d better be cautious when dealing with health care organizations” our study asked, “You ‘d better be cautious when dealing with health care providers”. The MMI formed a reliable scale in the present study (α = .92).

#### Vaccine necessity and concerns

Based on the work conducted by Pellowski and colleagues, study respondents were asked about their vaccine concerns and necessity [24]. To assess vaccine necessity, participants rated the extent to which they agreed with the following statements: “Without a vaccine, I am at high risk of dying from COVID-19,” “I need a vaccine to protect myself against COVID-19,” and “If I don’t get a vaccine, I could get very sick from COVID-19.” Participants also provided their level of agreement with three statements about vaccine concerns: “I worry about the long-term effects of the COVID-19 vaccine,” “I worry about COVID-19 having been adequately tested”, and “The idea of taking a COVID-19 vaccine worries me.” Both vaccine necessity (α = .89) and vaccine concerns (α = .88) formed reliable scales.

#### Vaccination intentions

Vaccination intentions, the primary dependent variable, were measured based on Fishbein and Ajzen’s recommendations [6]. Participants were asked to rate their responses to three questions on whether they will, intend to, and plan to get the COVID-19 shot. The three items formed a reliable scale (α = .98).

#### Demographics

Background information was collected for a variety of demographic factors. In addition to race, which was used to determine study eligibility, participants were asked about their age, sex, education, and income. Age was presented with a single open-ended response. Education was assessed using a 6-point scale ranging from 1 (less than high school) to 6 (advanced degree, e.g., Ph.D., JD). Income was assessed by asking participants about their entire household income in the previous year (2019). Participants responded by indicating which of 12 categories best described that income; the categories were in $10,000 increments (i.e., 1 = less than $10,000, 2 = $10,000–19,999 … 12 = more than $150,000). Additionally, participants were asked about their history of COVID-19 infection: whether they had received a positive COVID-19 diagnosis or know somebody who tested positive, or thought they had COVID-19. These demographic variables were examined as potential covariates.

### Data analysis

SPSS 25.0 was used to run the descriptive analysis and bivariate correlations for relevant variables. Using the statistical modeling program, Mplus Version 8 [40], we conducted structural equation modeling (SEM) analyses. The model goodness of fit was evaluated using the following criteria: the chi-square with a *p*-value greater than .05, realizing this measure can be a problematic metric due to sensitivity to sample size; a root mean square error of approximation (RMSEA) value less than .08; a value of standardized root mean squared residual (SRMR) less than .09; comparative fit index (CFI), of .90 or above, were considered acceptable fits [41, 42]. To assess the mediating effects, 95% confidence intervals based on 5000 bootstrap samples were computed. If the confidence interval for the mediating effect did not contain zero, this was evidence of an indirect effect via the mediator of interest [43].

## Results

### Demographics

The sample was comprised of an equal number of Black (*n* = 210) and White participants (*n* = 210). The average age of participants was 45.51 years (*SD* = 17.37). The sample was almost evenly split by sex; women comprised 51% of the sample. Most of the sample (69.2%) had less than a four-year college degree and reported making at least $50,000 (60.0%). Additionally, while only 23.2% of participants had, or suspected they had COVID-19, 60.9% of participants knew of someone who had or thought they had COVID-19.

### Bivariate relationships

In the full sample, trust in providers was positively associated with trust in healthcare information (*r* = .38, *p* < .001), negatively associated with medical mistrust in providers (*r* = −.16, *p* = .011), and positively associated with both vaccine necessity (*r* = .34, *p* < .001) and vaccine intentions (*r* = .41, *p* < .001). Additionally, there was no significant relationship between trust in providers and vaccine concerns (*r* = -.06, *p* = .21). Trust in healthcare information was negatively associated with mistrust of providers (*r* = −.23, *p* < .001). Additionally, it was positively associated with vaccine necessity (*r* = .36, *p* < .001), but negatively associated with vaccine concerns (*r* = −.11, *p* = .02). Trust in healthcare information was positively associated with vaccine intentions (*r* = .45, *p* < .001). Although mistrust in providers was not associated with vaccine necessity (*r* = −.05, *p* = .36), it was positively associated with vaccine concerns (*r* = .29, *p* < .001) and negatively associated with vaccine intentions (*r* = −.14, *p* = .004). Vaccine necessity and vaccine concerns were not significantly related (*r* = .03, *p* = .51). Finally, both vaccine necessity (*r* = .69, *p* < .001) and vaccine concerns (*r* = −.20, *p* < .001) were significantly related to vaccine intentions. Full descriptives and correlations appear in Table 1.

**Table 1:** Means, Sstandard Deviations, and Zero-order Correlation Matrix

	Descriptives		Bivariate Correlations					
Variable	M	SD	1	2	3	4	5	6
1. Trust in Providers	3.56	0.99	–					
2. Trust in Information	2.70	1.09	.38**	–				
3. Mistrust of Providers	3.21	1.02	−.16**	−.23**	–			
4. Vaccine Necessity	3.33	1.26	.34**	.36**	−.05	–		
5. Vaccine Concerns	3.61	1.19	−.06	−.11*	.29**	0.03	–	
6. Vaccine Intentions	3.53	1.52	.41**	.45**	−.14**	.69**	−.20**	–

*p < .05, **p < .01

### Confirmatory factor analysis

The model consisted of five latent variables: trust in providers, mistrust in providers, vaccine necessity, vaccine concerns, and vaccine intentions. Trust in healthcare information was an observed variable. Before testing measurement invariance, the fit of the model in both subsamples was tested [44]. The initial model did not provide an adequate fit for the Black sample, χ^2^(289, *n* = 210) = 786.45, *p* < .001; CFI = 0.87, RMSEA = 0.09 (90% CI: .08, .10), SRMR = 0.12. There were three reverse-coded items as part of the measure of trust that did not have factor loadings above .40 and were removed as a result [45]. The removal of these items bettered fit, χ^2^(220, *n* = 210) = 402.76, *p* < .001; CFI = 0.95, RMSEA = 0.063 (90% CI: .05, .07), SRMR = 0.05. A subsequent examination of modification indices indicated two trust items reflecting doctors’ honesty and thoroughness should be correlated. This resulting model provided adequate fit, χ^2^(219, *n* = 210) = 380.56, *p* < .001; CFI = 0.96, RMSEA = 0.06 (90% CI: .05, .07), SRMR = 0.05.

Similarly, the initial model did not provide adequate fit for the White sample, χ^2^(289, *n* = 210) = 1051.86, *p* < .001; CFI = 0.84, RMSEA = 0.11 (90% CI: .11, .12), SRMR = 0.15. As with the Black sample, the reverse-coded items did not have factor loadings above .40 and were removed. The resulting measure resulted in an improved model, χ^2^(220, *n* = 210) = 501.44, *p* < .001; CFI = 0.94, RMSEA = 0.08 (90% CI: .07, .09), SRMR = 0.06. Modifications were again made, correlating error terms of two sets of items – trust: doctors’ honesty and thoroughness and necessity: risk of dying or getting very sick without the vaccine – resulting in a model with adequate fit, χ^2^(218, *n* = 210) = 405.20, *p* < .001; CFI = 0.96, RMSEA = 0.06 (90% CI: .05, .07), SRMR = 0.06.

### Measurement invariance

Configural invariance was tested first, which determines whether the same items measure the same constructs across groups. This model demonstrated good fit, χ^2^(437, *n* = 420) = 785.76, *p* < .001; CFI = 0.96, RMSEA = 0.06 (90% CI: .06, .07), SRMR = 0.05. Next, metric invariance was assessed by comparing the fit of the metric model to the fit of the configural model. This level of measurement invariance examines whether the factor loadings for items are equivalent across groups – an indication that constructs have the same meaning across groups [46]. The model for metric invariance produced a significant change in chi-square, χ^2^(455, *n* = 420) = 820.53, *p* < .001; CFI = 0.95, RMSEA = 0.06 (90% CI: .06, .07), SRMR = 0.06. Modification indices were then examined to ascertain whether partial metric invariance could be obtained [47]. By freeing the factor loading of one trust item (i.e., “Your doctor is extremely thorough and careful”), a model was produced with a non-significant change in chi-square, χ^2^(454, *n* = 420) = 809.48, *p* < .001; CFI = 0.95, RMSEA = 0.06 (90% CI: .05, .07), SRMR = 0.06.

Using this partially invariant metric model [48], scalar invariance was examined, which tests constrained intercepts. The scalar model produced a significant change in chi-square, χ^2^(471, *n* = 420) = 838.03, *p* < .001; CFI = 0.95; RMSEA = 0.06 (90% CI: .05, .07), SRMR = 0.06. There was only one intercept that could be freed to improve fit (i.e., “The idea of taking a COVID-19 vaccine worries me”). Freeing this intercept, resulted in a model with a nonsignificant change in chi-square, χ^2^(470, n = 420) = 827.91, *p* < .001; CFI = 0.96; RMSEA = 0.06 (90% CI: .05, .07), SRMR = 0.06. Thus, the data indicated that there was partial invariance across Black and White participants.

### Structural model

**Fig. 1:**
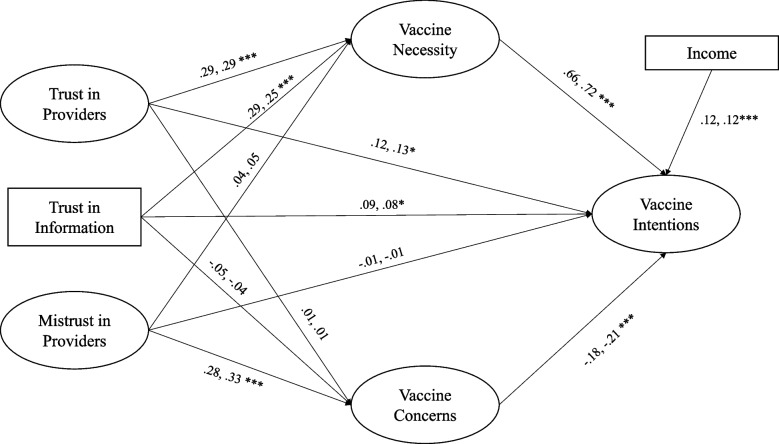
Structural model. *Note*. The figure shows the relationships and standardized pathway coefficients for the final, fully constrained model. The standardized coefficient for Black participants is presented first, followed by the standardized coefficient for White participants. *p ≤ .05, **p ≤ .01, ***p ≤ .001

This partially invariant measurement model was used to examine the structural relationships. First, a baseline model was tested; in this model, all pathways are freed. The baseline model revealed that two demographic variables may be covariates – age and income. Thus, these were the only variables included in the resulting analyses. The baseline model produced adequate fit, χ^2^(600, *n* = 420) = 1040.38, *p* < .001; CFI = 0.95, RMSEA = 0.06 (90% CI: .05, .07), SRMR = 0.07. Next, a fully constrained model was tested. This model produced adequate fit and a non-significant change in chi-square, χ^2^(616, *N* = 420) = 1063.48, *p* < .001; CFI = 0.95, and RMSEA = 0.06 (90% CI: .05, .07), SRMR = 0.08. Thus, the models are structurally invariant. There are no differences in the pathways for Black and White participants. The final model appears in Fig. 1.

Trust in providers was associated with vaccine necessity (*b* = .36, *p* < .001) but not vaccine concerns (*b =* .01*, p =* .94). Similarly, trust in information was associated with vaccine necessity (*b* = 0.27, *p* < .001) but not vaccine concerns (*b* = −.04, *p* = .51). Mistrust of providers, however, was significantly related to vaccine concerns (*b* = 0.34, *p* < .001), but not vaccine necessity (*b* = .05, *p* = .39). Trust in providers (*b* = 0.21, *p* = 0.04) and trust in information (*b* = 0.12, *p* = 0.03), were significantly related to intentions to get the COVID-19 vaccine. Mistrust in providers was not significantly related to vaccine intentions (*b* = −.01, *p* = .88). Vaccine necessity (*b* = 0.96, *p* < 0.001) and vaccine concerns (*b* = − 0.27, *p* < 0.001), along with income (*b* = 0.05, *p* < 0.001) were also significantly related to COVID-19 vaccine intentions. Structural model information appears in Table 2.

**Table 2:** Parameter estimates

Pathway	b	B_B_	B_W_	SE	p	95% CI
Vaccine Necessity						
Trust in Providers	0.36	0.29	0.29	0.08	<.001	[.20, .51]
Trust in Information	0.27	0.29	0.25	0.05	<.001	[.16, .37]
Mistrust in Providers	0.05	0.04	0.05	0.06	0.39	[−.07, .17]
Vaccine Concerns						
Trust in Providers	0.01	0.01	0.01	0.08	0.94	[−.15, .16]
Trust in Information	− 0.04	− 0.05	− 0.04	0.06	0.51	[−.16, .08]
Mistrust of Providers	0.34	0.28	0.33	0.07	<.001	[.21, .47]
Vaccine Intentions						
Trust in Providers	0.21	0.12	0.13	0.10	0.04	[.02, .41]
Trust in Information	0.12	0.09	0.08	0.06	0.03	[.01, .22]
Mistrust of Providers	−0.01	−0.01	−0.01	0.07	0.88	[−.15, .13]
Vaccine Necessity	0.96	0.66	0.72	0.08	< .001	[.80, 1.12]
Vaccine Concerns	−0.27	−0.18	−0.21	0.06	< .001	[−.39, −.15]
Age	−0.01	−0.06	− 0.06	0.003	0.10	[−.01, .001]
Income	0.05	0.12	0.12	0.02	<.001	[.02, .08]

As the variance differed among populations, the standardized coefficients for Black and White participants are not always equivalent. b = unstandardized coefficient; B_B_ = standardized coefficient for Black participants; B_W_ = standardized coefficient for White participants; *SE* Standard error, CI Confidence interval

### Indirect effects

An examination of confidence intervals revealed that trust in providers had a direct effect on vaccine intentions (95% CI: .02, .41). Additionally, it exerted indirect effects on vaccine intentions through vaccine necessity (95% CI: .19, .50). This pattern held for trust in healthcare information as well; there was both a direct effect (95% CI: .01, .22) and an indirect effect through vaccine necessity (95% CI: .16, .36). Mistrust of providers had no direct effect on vaccination intentions (95% CI: −.15, .13) but did have an indirect effect on vaccination intentions through vaccine concerns (95% CI: −.15, −.03). There were no other significant direct or indirect effects; full information is displayed in Table 3.

**Table 3:** Direct and indirect effects

Association	b	B_B_	B_W_	SE	p	95% CI
Trust in Providers						
Direct Effect on Vaccine Intentions	0.21	0.12	0.13	0.10	0.04	[.02, .41]
Indirect Effect via Vaccine Necessity	0.34	0.19	0.21	0.08	<.001	[.19, .50]
Indirect Effect via Vaccine Concerns	−0.002	−0.001	− 0.001	0.02	0.94	[−.04, .04]
Total Indirect Effect	0.34	0.19	0.21	0.08	<.001	[.19, .50]
Total	0.55	0.30	0.33	0.11	<.001	[.35, .76]
Trust in Provider Information						
Direct Effect on Vaccine Intentions	0.12	0.09	0.08	0.06	0.03	[.01, .22]
Indirect Effect via Vaccine Necessity	0.26	0.19	0.18	0.05	<.001	[.16, .36]
Indirect Effect via Vaccine Concerns	0.01	0.01	0.01	0.02	0.52	[−.02, .04]
Total Indirect Effect	0.27	0.20	0.19	0.05	<.001	[.16, .37]
Total	0.38	0.29	0.27	0.07	<.001	[.25, .52]
Mistrust of Providers						
Direct Effect on Vaccine Intentions	−0.01	−0.01	−0.01	0.07	0.88	[−.15, .13]
Indirect Effect via Vaccine Necessity	0.05	0.03	0.04	0.06	0.40	[−.06, .16]
Indirect Effect via Vaccine Concerns	−0.09	−0.05	− 0.07	0.03	0.002	[−.15, −.03]
Total Indirect Effect	−0.04	−0.02	− 0.03	0.06	0.47	[−.16, .07]
Total	−0.05	−0.03	− 0.04	0.07	0.47	[−.20, .09]

As the variance differed among populations, the standardized coefficients for Black and White participants are not always equivalent. b = unstandardized coefficient; B_B_ = standardized coefficient for Black participants; B_W_ = standardized coefficient for White participants; *SE* Standard error, CI Confidence interval

## Discussion

The current study set out to examine the relationships between trust in healthcare providers, trust in information, mistrust of healthcare providers, vaccine concerns, vaccine necessity, and COVID-19 vaccine intentions. More specifically, vaccine concerns and vaccine necessity were examined as potential mediators of the relationships between trust and mistrust and COVID-19 vaccine intentions among Black and White Americans. The model fit the data for Black and White Americans, with vaccine necessity and vaccine concerns mediating some of the relationships between trust in providers, trust in provider information, and mistrust of providers on intentions. This suggests that these relationships, which are important in other contexts, are also relevant for COVID-19 and align with other work suggesting psychological factors (e.g., vaccine benefits, adverse outcomes from vaccination) influence vaccination behavior [49, 50].

There was a direct relationship between vaccination intentions for both trust in providers and trust in healthcare information. Previously, these facets have been collapsed. In the current study, the two concepts were not so highly correlated to suggest overlapping concepts (*r* = .38, *p* < .01). Additionally, an examination of the standardized coefficients suggests this relationship was stronger for trust in providers. This may indicate that individuals make distinctions between their perceptions of providers and the healthcare information they provide. According to theories like the elaboration likelihood model and the heuristic systematic model, individuals process information centrally (e.g., by carefully evaluating message arguments) and/or peripherally (e.g., by examining cues in the message) [51–53]. The data for this study were collected prior to vaccines being widely available and the public was highly concerned about the pandemic [54]. Amidst those circumstances, individuals are likely motivated and thus more likely to engage in central processing; however, the amount of information and changing recommendations may create a situation in which the ability to process the message is lowered, resulting in peripheral processing. Thus, while both can influence vaccine intentions, they may be operating via different mechanisms; for instance, healthcare professionals might serve as a source cue of credible information [55]. Future work should examine information processing and the effects of source cues on vaccination willingness for COVID-19 vaccination.

Consistent with previous work [24], trust in healthcare providers and trust in healthcare information exerted indirect effects on vaccine intentions through vaccine necessity. This relationship existed for both Black and White participants. Contrary to our hypotheses, neither trust in providers nor trust in healthcare information, however, influenced vaccine concerns. In other contexts, trust in providers appeared to mitigate concerns [24]. In the context of the COVID-19 vaccine, the hope would have been that information from providers would not only increase the perceived necessity of vaccines but also allay concerns about the vaccine. This may suggest that the information providers were giving or how it was being given were not effective at mitigating safety concerns. Alternatively, the information provided to the public regarding vaccine concerns at the time of data collection came from a variety of sources, much broader than solely physicians. A great deal of information surrounding the safety of the COVID-19 vaccine emanated from public health officials at the national, state, and local levels. In all, much of the information the public received at the time may not have been coming healthcare providers; calls in April 2021 for more messaging from clinical physicians suggest this may have been the case [56]. Healthcare providers, however, are likely the messengers best situated to address both vaccine concerns and necessity as they have been the most trusted sources for COVID-19 information and across countries has been found to be one of the strongest predictors of vaccination [23, 57].

On the other hand, COVID-19 may simply be a different context; previous work that found a significant, negative relationship between trust in providers and concerns was in the context of ART [23]. This is a context in which individuals are discussing the treatment of a diagnosed condition. Thus, it may be more reasonable for providers to be discussing and addressing concerns. This varies from COVID-19 in which the vaccine is a preventive measure and communication about the vaccine comes from sources more widely. It is also possible that the speed that the public perceives related to the vaccines’ production is too heightened for the disparate, non-specific communication to be effective.

Additionally, mistrust in providers exerted indirect effects on vaccine intentions via vaccine concerns. In other words, medical mistrust does decrease behavioral intentions related to COVID-19 vaccination through vaccination concerns. Medical mistrust, however, did not negatively impact beliefs about vaccine necessity, nor did it have a direct effect on vaccination intentions. Recent work has found a relationship between mistrust and vaccine intentions [58], suggesting a direct effect might exist. The findings of that study could be due to their broader conceptualization of medical mistrust, which encompassed both distrust of providers and distrust in the vaccine; here, these concepts are separated by medical mistrust and vaccine concerns. Our findings suggest that this separation may point to a more nuanced understanding of the relationship between medical mistrust and vaccination intentions. Medical mistrust may only influence vaccination intentions insofar as it impact vaccination concerns. Recent qualitative work has suggested that concerns about the efficacy and safety of the vaccine links medical mistrust and intentions to be vaccinated [59]. Further work to understand the connections between medical mistrust and vaccination intentions is necessary.

These findings suggest that while information from providers influences individuals’ perceived necessity of vaccines, it fails to address individuals’ perceived concerns about vaccines. Vaccine necessity was shown to be more strongly associated with intentions than vaccine concerns, suggesting that one route to overcoming vaccine hesitancy is to increase perceptions of the vaccine’s necessity. Even today, many Americans doubt the necessity and efficacy of getting vaccinated; only 50.8% of Americans are currently fully vaccinated [60]. Though vaccine necessity appears to play a significant role in vaccination intentions, vaccination concerns must also be addressed; research conducted a few months before this study found that “intenders” (i.e., those who intended to get the vaccine) were more likely to believe in the safety of the vaccine (i.e., have lowered vaccine concerns) [61]. To improve vaccine uptake, existing research suggests that pro-vaccine messaging should validate the hesitancy of individuals and frame the necessity of the vaccine around risks associated with the virus [62]. Our results suggest communication efforts should acknowledge individuals’ concerns, and perhaps their mistrust, in addition to increasing perceptions of vaccine necessity. These recommendations echo those made as a result of a comprehensive review of literature on vaccine hesitancy among Black American and Hispanic/Latinx communities [63]. Ultimately, leveraging trust and addressing mistrust to influence individuals’ perceptions about vaccines may prove to be an important step in improving vaccine uptake among many US communities.

Notably, the model was found to be structurally invariant, suggesting that the pathways did not differ for Black and White Americans. While trust and mistrust are known to vary among various segments of the population, including Black and White Americans, the current study suggests that once that trust or mistrust is established, the relationships between those variables and vaccination intentions do not vary between Black and White Americans. Caution should be taken in applications of what this means for messaging. It is possible that these particular measures reflect commonalities in perceptions about the vaccine. For instance, with vaccine concerns, the items reflect worries about adequate testing, long-term effects, and worries about the vaccine. These items may not capture how vaccine concerns manifest differently for persistently marginalized populations, like Black Americans. The reasons Black Americans have worries about the vaccine could differ from White Americans; while responses to the items may be similar, the reasons for those beliefs may differ. Further work in this area would be illuminating and may allow for a deeper understanding of relationships among these constructs. Even though individuals, or groups, may be mistrustful or have concerns, the beliefs underlying those perceptions may differ. As a result, communication will need to differ and explicitly target those underlying beliefs to be effective [64]. One way to approach this may be to utilize the ASPIRE framework in which initiating discussions about concerns, and thereby ascertaining specific beliefs, and answer those questions and concerns is built into the process of discussing vaccination with individuals [65].

### Limitations and future directions

The current findings must be considered in the context of their limitations. First, this was a cross-sectional survey conducted in January 2021; thus, despite the order of the variables tested being based on empirical evidence, our findings cannot speak directly to causality. To accumulate further support for these relationships, longitudinal data would be necessary. Additionally, January 2021 was a very specific time point in the arc of the pandemic response; vaccines had just received emergency use authorization in December 2020 (Pfizer) and January 2021 (Moderna) and were still widely unavailable. While the timing may have been opportune for measuring vaccination intentions, it was also a period filled with uncertainty and lingering questions as the country awaited additional information about vaccine safety and government’s vaccination strategy. As time has passed, in which more data was collected and individuals have been able to observe the experiences of others, there may be less concerns around vaccine safety leading to increased vaccine acceptance [66]. Empirical work is necessary to determine the extent to which these relationships are stable over time.

It should also be noted that the present study asked about trust and mistrust generally. Trust and mistrust, however, can have varying objects [13, 67]; one dimension on which the object can vary is the individual-system. Here, the object is system level (i.e., providers, generally). It is possible that trust and mistrust in one’s own provider (i.e., individual level) may follow a different pattern. Subsequent work should examine whether these patterns hold for trust in one’s own physician. Studies specifically examining in trust in one’s own physician have found significant relationships between that trust and likelihood of vaccination [12, 68]. Perhaps, trust in one’s own provider or trust in provider information from one’s own provider could ameliorate concerns about the vaccine. We must note, however, that whether trust in one’s own provider ameliorates or heightens concern may depend on the healthcare professionals’ attitudes toward the vaccine [69]. Furthermore, future work could build upon work involving trust in scientists and trust in government entities to examine the relationships between these objects and trust and vaccine necessity and concerns [16, 70].

In the current study, trust in health information was measured regarding trust in healthcare providers. Recent work has found that the source of the information has differential effects on COVID-19 behaviors [16, 71]. Thus, future work in this area should test trust in other sources of health information in conjunction with vaccine necessity, concerns, and intentions. Additionally, further work could expand upon or extend these findings to other vaccinations beliefs. While vaccine necessity and concerns, as conceptualized in the present study, are consistent with how other scholars have investigated similar constructs, this represents a small set of possible vaccination beliefs. Individuals hold an array of identities or experiences that impact these factors and are embedded in a larger ecological context. It would be prudent for scholars to examine the relationships between trust, mistrust, vaccination intentions, and other vaccination beliefs (e.g., getting the vaccine would protect others in your community) and how these relationships may differ among various sociodemographic groups [72, 73]. As we do so, it will be important to also capture the processes we think cause differences among demographic groups (e.g., racism, experiences of discrimination) as opposed to group membership (e.g., race).

Finally, it must also be noted that while vaccination intentions were the outcome of interest, vaccination intentions do not always equate to vaccination behaviors [74]. Even if individuals are willing to be vaccinated there can be barriers to enacting that behavior (e.g., the ability to take off work to receive the vaccine or deal with side effects) [58]. Furthermore, there may be a feedback loop such that difficulty accessing the vaccine or hearing about others’ difficulty accessing the vaccine can increase medical mistrust [34, 75], resulting in a decreased likelihood of getting vaccinated. Thus, efforts must not only account for vaccine-relevant perceptions but must be done in tandem with structural efforts to promote access and equity in vaccine distribution. Efforts to a) ensure equity and access and b) not only listen to but also be responsive to community concerns (including, but not limited to those around the vaccine) can serve to not only reduce barriers but also facilitate trust building [76]. As we continue to understand the nuances of the relationships between trust, mistrust, and vaccination necessity, concerns, and beliefs, we cannot lose sight of the environment and context these perceptions are situated within.

These issues are indicative of a larger point – the present study and its findings are only a starting place. While our suggestions for communication that understands and is responsive to individuals’ beliefs align with recommendations of other scholars [65, 69], this is truly a single component of what should be a broader, multi-level approach. While these beliefs contribute to intentions, Messaging, alone, will never been the solution, nor is it the best strategy in all situations. One potential framework for assessing where efforts should be focused, and under what circumstances, is the stages of change model. This model, often used to tailor behavior change persuasive efforts, takes into consideration where people are in their decision-making process (e.g., have they begun to think about the behavior or are they close to engaging in the behavior?). Recent work under this framework has posited the for those who are vaccine receptive (i.e., “vaccine hesitant” individuals who will accept vaccination if it requires minimal effort), structural issues like access are critical for vaccine acceptance [77]. It may be the case that communication efforts around the relationships examined in the present study are best suited for individuals who are precontemplative (i.e., not yet considering engaging in the behavior). Ultimately, additional studies will need to explore whether this bares out and other practical solutions to addressing these concerns.

## Conclusion

In addressing vaccine uptake, the factors underlying vaccine hesitancy and how they may differ across groups must be considered. The need remains to be committed and intentional about building trust and addressing mistrust between individuals and the medical system. As we continue to address these factors, we must not assume that practices or messages that influence trust will inherently impact mistrust as these are constructs that have differential impacts on outcomes. Additionally, as vaccination rates slow [78], it will be crucial for communication to not only demonstrate the necessity of vaccines but also address concerns; this is likely a task for all healthcare professionals, from physicians to pharmacists. There is a wide range of concerns about the vaccine [11, 79]; thus, to truly address individuals’ concerns, specific concerns must be elicited and addressed. Ultimately, ensuring effective communication, particularly that communication that a) addresses the necessity of vaccines as well as the beliefs underlying vaccine concerns and b) repairs trust with the public, may aid continued efforts to address vaccine hesitancy.

## Data Availability

Data for this study is available upon request by contacting the corresponding author.
